# Hyperbaric Oxygen Promotes Proximal Bone Regeneration and Organized Collagen Composition during Digit Regeneration

**DOI:** 10.1371/journal.pone.0140156

**Published:** 2015-10-09

**Authors:** Mimi C. Sammarco, Jennifer Simkin, Alexander J. Cammack, Danielle Fassler, Alexej Gossmann, Luis Marrero, Michelle Lacey, Keith Van Meter, Ken Muneoka

**Affiliations:** 1 Department of Cell and Molecular Biology, Tulane University, New Orleans, Louisiana, United States of America; 2 Department of Mathematics, Tulane University, New Orleans, Louisiana, United States of America; 3 Department of Medicine, Louisiana State University Health Sciences Center, New Orleans, Louisiana, United States of America; 4 Department of Veterinary Physiology & Pharmacology, Texas A&M University, College Station, Texas, United States of America; University of Connecticut Health Center, UNITED STATES

## Abstract

Oxygen is critical for optimal bone regeneration. While axolotls and salamanders have retained the ability to regenerate whole limbs, mammalian regeneration is restricted to the distal tip of the digit (P3) in mice, primates, and humans. Our previous study revealed the oxygen microenvironment during regeneration is dynamic and temporally influential in building and degrading bone. Given that regeneration is dependent on a dynamic and changing oxygen environment, a better understanding of the effects of oxygen during wounding, scarring, and regeneration, and better ways to artificially generate both hypoxic and oxygen replete microenvironments are essential to promote regeneration beyond wounding or scarring. To explore the influence of increased oxygen on digit regeneration *in vivo* daily treatments of hyperbaric oxygen were administered to mice during all phases of the entire regenerative process. Micro-Computed Tomography (μCT) and histological analysis showed that the daily application of hyperbaric oxygen elicited the same enhanced bone degradation response as two individual pulses of oxygen applied during the blastema phase. We expand past these findings to show histologically that the continuous application of hyperbaric oxygen during digit regeneration results in delayed blastema formation at a much more proximal location after amputation, and the deposition of better organized collagen fibers during bone formation. The application of sustained hyperbaric oxygen also delays wound closure and enhances bone degradation after digit amputation. Thus, hyperbaric oxygen shows the potential for positive influential control on the various phases of an epimorphic regenerative response.

## Introduction

Oxygen has long been known to be a key player in both bone repair and bone development, and we have recently shown that a dynamic oxygen environment is critical for optimal bone regeneration associated with an epimorphic regenerative response [1]. While axolotls and salamanders have retained the ability to regenerate whole limbs, mammalian regeneration is restricted to the digit tip [2–6] in mice [4, 5], primates, and humans [7–10]. In mice this multi-tissue regenerative model provides a predictable phase progression of regeneration. After amputation of the distal tip of the third phalangeal element (P3) there is an initial bone degradation phase, followed by wound closure, blastema formation, and finally redifferentiation of the blastema into bone with surrounding soft tissue compartments [1, 4, 5, 11]. This regenerative model provides an excellent opportunity to more closely study the influence of oxygen during bone regeneration.

Our previous study revealed that local oxygen tension is a dynamic event that is temporally influential in degrading and rebuilding bone [1]. Given that regeneration is dependent upon a specific changing oxygen environment, a better understanding of the effects of oxygen during wounding, scarring, and regeneration and the ability to artificially generate both hypoxic and oxygen replete microenvironments is essential for optimal replacement of bone and tissue. A higher partial pressure of oxygen is required to generate an elevated oxygen diffusion gradient for oxygen delivery to surrounding tissue. Oxygen moves down a gradient after entering the lungs from a partial pressure of about 160 mmHg in the atmosphere to less than 1 mm Hg in the mitochondria [12]. The delivery of oxygen under pressure, or hyperbaric oxygen (HBO) treatment, allows for the systemic oxygen super-saturation of plasma, elevating the amount of dissolved oxygen in blood *in vivo*, above the maximal capacity of hemoglobin [13]. The application of HBO, in turn, creates a dynamic oxygen environment that has short- and long-term effects within the applied system. Typically, clinical application of HBO is a daily event that is sustained for a week or more. Tissue oxygen studies in mice show that sustained HBO treatment reduces peak oxygen exposure levels *in vivo* over time [14, 15], and that tissue oxygen levels return to baseline much more quickly as treatment progresses [15]. These oscillating whole-tissue oxygen levels in turn have wide-reaching effects at a cellular and molecular level. This makes the timing, application, and frequency of HBO three independent influences *in vivo*. We hypothesize that the influence of HBO is specific to individual wound models, and this finding illustrates the critical need to differentiate between continuous application and HBO applied in a discrete and temporally specific manner.

We recently showed that two single HBO treatments, applied during a hypoxic phase of regeneration extends the degradation phase and attenuates the bone regeneration phase [1]. This finding illustrates a strong argument for a closer investigation of the timed relationship between HBO and regenerative events *in vivo*. Here we expand upon those findings, detailing the influence of HBO applied twice a day throughout P3 regeneration *in vivo*. The data show that while the clinical application of HBO enhances the degradation phase and delays wound closure over the bone stump, sustained oxygen-mediated degradation also confers regenerative capacity to a much more proximal site on P3 than previously thought, facilitates a proximal blastema structure, and results in more organized bone formation. Our data indicate that the use of pressurized oxygen to manipulate the stages of regeneration shows promise and warrants further investigation in future studies.

## Results

### Daily HBO treatment enhances bone degradation

**Fig 1:**
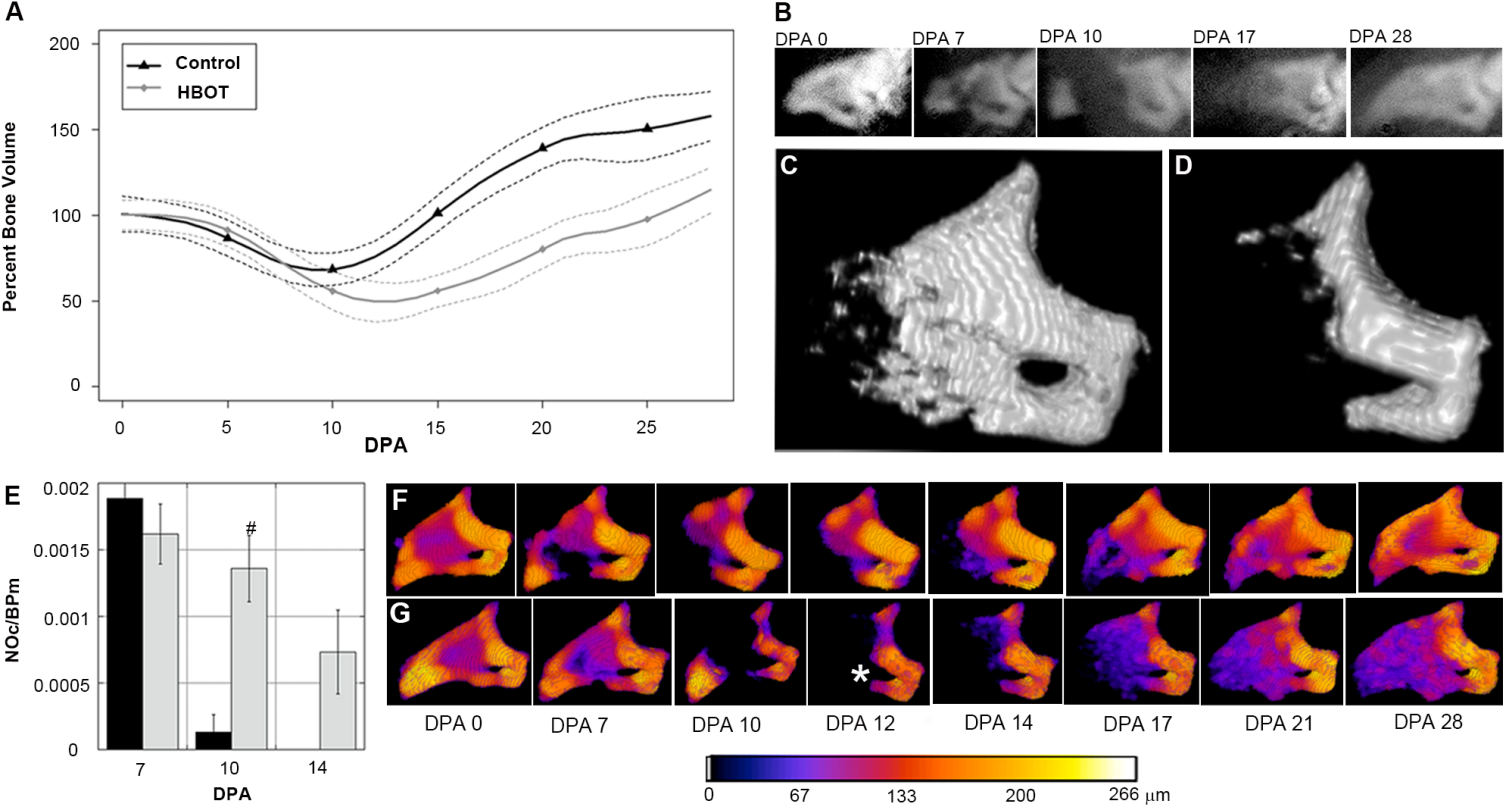
Continuous HBO treatment enhances bone degradation. (A) Bone regeneration during HBO application shows a similar rate of bone degradation before beginning to grow bone at 12 DPA (N = 4 mice, N = 16 digits). Samples were analyzed for bone growth using μCT. Data are normalized to initial 0 DPA bone volume and analyzed using a SS ANOVA algorithm accounting for variation between the individual mice (see methods). (B) Representative X-ray images of a regenerating P3 digit at 0, 7, 10, 17, and 28 DPA. (C) 14 DPA time point capture showing the P3 regenerative response in a control digit, (from A). (D) 14 DPA time point capture of a representative digit (from A) treated with continuous HBO twice daily showing increased bone degradation. Full time lapse sequence available in supplemental materials. (E) Effect of daily HBO application on osteoclast numbers at 7, 10, and 14 DPA. Results are expressed as mean ± SEM. ^#^ P<0.05, comparison of control to HBO. NOc/BPm; number of osteoclasts/bone perimeter. (F) Micro-CT scans of the same control sample seen in C and (G) same HBO sample seen in D are pseudo-colored according to trabecular thickness. (*) Asterisk indicates degradation through the proximal os-hole. Color changes indicate bone thickness in μm.

Mouse digits were amputated as described previously [1] and mice were administered 100% oxygen at 2.4 ATA for 90 minutes. This treatment was applied three times a day for the first two days following amputation and twice daily for the duration of the experiment. This daily HBO treatment regime is considered to be similar to clinically applied Undersea Hyperbaric Medical Society (UHMS) standard HBO treatment in humans. Bone volume for control and daily HBO treatment groups was quantified using Micro- Computed Tomography (μCT) and plotted against time following digit amputation. The regeneration of control (untreated) digits was characterized by a series of stages that included bone degradation (histolysis), epidermal closure, blastema formation and redifferentiation [1, 5, 16]. Changes in bone volume in control digits during the regeneration response showed a decline associated with histolysis that transitioned to a rapid bone rebuilding phase. While this general trend in bone volume change was observed in HBO treated digits, the timing of the transition from degradation to rebuilding was highly variable (S1A Fig). This is best seen when the bone volume data are analyzed using Smoothing Spline Analysis of Variance (SS ANOVA) models to predict continuous changes in volume for control versus HBO treated regenerates (Fig 1A). The use of SS ANOVA provides an independent statistical analysis that validates traditional *t*-tests at individual time points (S1B Fig), accounts for repeated sampling, and enables prediction of expected bone volume changes at all experimental time points. The fitted SS ANOVA model also enables the estimation of continuous confidence intervals that establish useful limits of differences resulting from the treatment effects. The analysis of daily HBO-treated regenerates showed that treated digits begin the initial stages of regeneration in parallel with controls but deviate during later stages of the response (Fig 1A). Representative X-ray images for control digits are shown at 0, 7, 10, 17, and 28 days post-amputation (DPA) for visual comparison (Fig 1B).

Mice treated with daily HBO treatment showed an enhanced level of bone loss which reached statistical significance between 10 DPA and 15 DPA (Fig 1A and 1D). After amputation, the bone degrades. By 12 days amputation with HBO treatment, mice lost an average of 50% of the day 0 bone volume. In contrast, mice without HBO treatment only lost an average of 30% of the day 0 bone volume. On average, control digits transition from a bone loss to a bone building stage around 9 DPA. This transition occurs at a later time point in HBO treated digits. This difference in bone loss is clear from μCT rendering of digits at 14 DPA (Fig 1C and 1D). Spatiotemporal renderings showing changes in bone morphology during the regenerative response were created and can be viewed in S1 and S2 movies. The quantitative analysis of bone volume changes in control and HBO treated digits (Fig 1A) emphasizes both the total amount of bone loss and changes in the timing of the degradation phase. These results clearly show that HBO treated digits display a delayed transition from bone degradation to bone regrowth when compared to control mice. Our data suggest that while the overall rate of degradation does not differ from controls, the period of degradation is prolonged and extends to a more proximal level, enhancing the total amount of bone loss. Similarly, after transitioning from the degradation phase to the regrowth phase, daily HBO-treated regenerates displayed a rate of new bone formation that parallels that of controls.

Bone degradation is the first discernable anatomic phase of P3 regeneration and culminates with the endogenous re-amputation of the bone stump by osteoclast activity. Bone degradation is followed by epithelial wound closure through the bone gap created between the remaining stump and the ejected bone [5]. Histological analysis of degradation quantifying the number of osteoclasts as a function of total bone perimeter (NOc/BPm), where osteoclasts were defined by being multinucleated, a ruffled edge border, and localization within either a resorptive bone pit or directly adjacent to the bone surface, showed osteoclasts on both periosteal and endosteal bone surfaces. Quantification of osteoclasts showed that while the number of osteoclasts at 7 DPA were initially comparable between control and daily HBO treated digits, at 10 DPA osteoclast numbers declined in control digits (2/8 samples had osteoclasts) and were completely absent by 14 DPA. In contrast, daily HBO treated digits maintained high numbers of osteoclasts at 10 DPA (8/8 samples had osteoclasts) and were still observed at 14 DPA (6/8 samples had osteoclasts) (Fig 1E). Our data show that the degradation is injury specific given that no degradation was seen in the unamputated digits subjected to HBO treatment (S2 Fig). These data are consistent with μCT measurements of bone volume showing HBO treatment extends the bone degradation phase thereby delaying the transition to bone regrowth during the regeneration process.

The spatial regulation of bone resorption in oxygen-mediated degradation versus controls also appeared to show differences. Analysis of bone thickness (trabecular thickness) using μCT 3D renderings of both control (Fig 1F) and HBO treated (Fig 1G) digits showed evidence of bone pits on the external surface of the control digit at 7 DPA that are not evident on the HBO treated digit. This observation suggests that HBO treatment differentially suppresses osteoclast activity outside of the marrow cavity. This oxygen-mediated degradation also extended bone regression in 9 of 16 digits to a more proximal level resulting in the degradation of the ventrally located os-hole, a foramen specific to the P3 phalanx [5], as well as completely eliminating a definitive P3 bone marrow cavity (Fig 1G, asterisk, 12 DPA).

### HBO treatment delays wound closure and blastema formation

**Fig 2:**
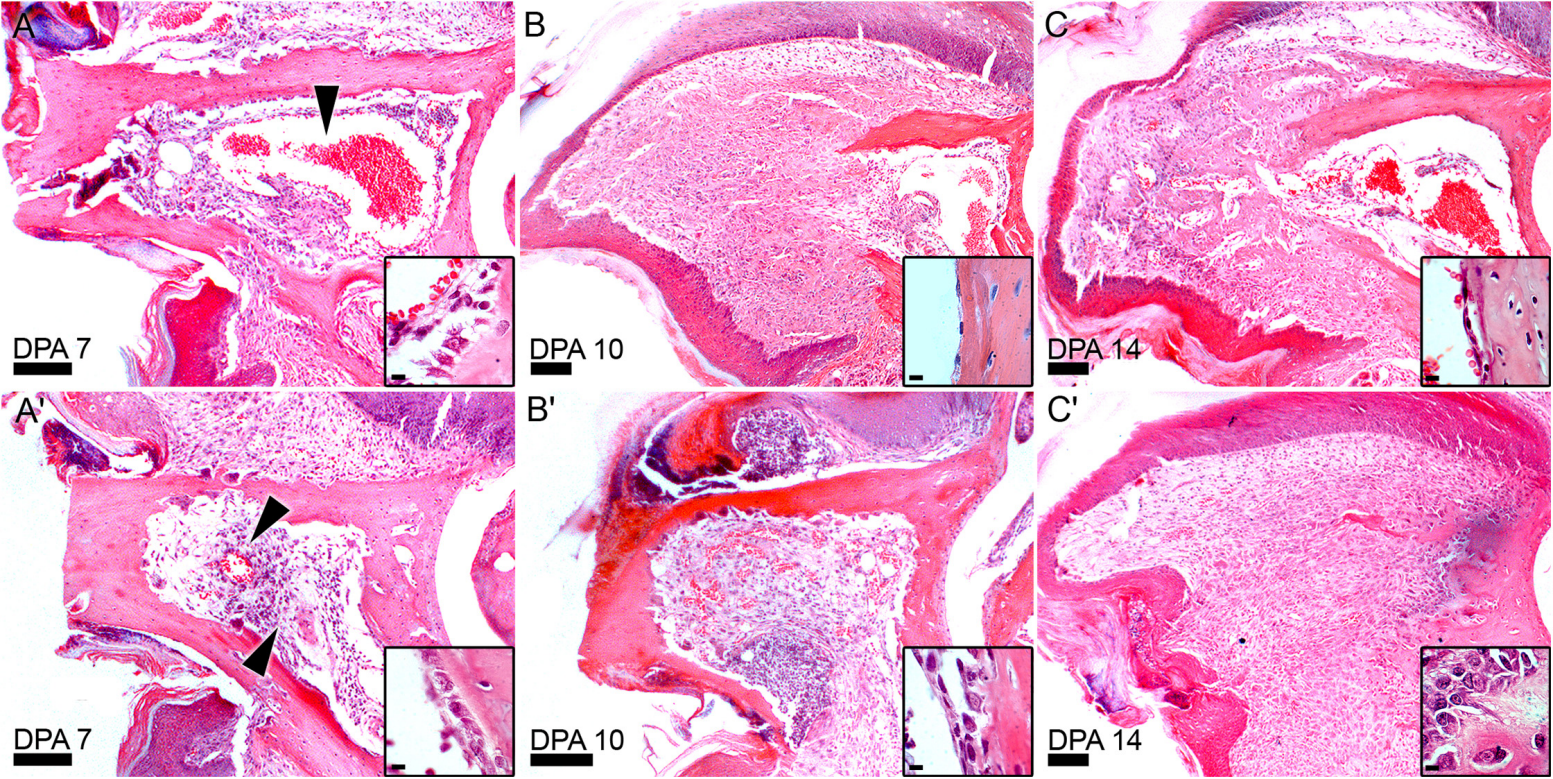
HBO treatment delays wound closure and blastema formation. H&E staining of 7, 10, and 14 DPA sections of control (A-C) and HBO-treated (A'-C') digits show cellular changes in the marrow and delayed wound closure and blastema formation in HBO-treated digits. Arrows indicate areas of vasculature on control 7 DPA (A) and HBO treated 7 DPA (A') samples. (B') Epidermis remains open at 10 DPA in samples treated with hyperbaric oxygen. (C') Blastema formation is delayed until 14 DPA in HBO-treated samples when control samples (C) are already forming bone. Higher magnification analysis of the proximal endosteum show activated osteoblasts in HBO-treated samples (A'-C') while control samples return to an inactivated morphology at 10 DPA (B). Scale bars = 100 μm in panels and 5 μm in insets. (N = 8 HBO and N = 7 control with representative figure shown).

The degradation phase of digit tip regeneration typically ends around 10 DPA and is associated with the closure of the wound epidermis [5]. Wound closure itself is thought to be essential for the regenerative response [17], and linked to the transition from the histolytic phase to blastema formation [18]. Since the degradation period is extended in HBO treatment, the timing of wound closure was analyzed. Complete wound closure is a clear-cut event easily distinguished by analyzing serial histological sections of the amputated stump. Fig 2 contains representative images of the time course of regeneration of a control digit (Fig 2A–2C) and in an animal treated with HBO (Fig 2A'–2C'). Wound closure analysis showed that HBO treatment negatively influenced wound closure after digit amputation compared to controls. When analyzed at 10 DPA (Fig 2B and 2B'), only 50% of HBO treated digit wounds were closed (4/8) compared to 100% of control digit wounds (10/10). While wound closure was complete in the majority of HBO treated digits at 14 DPA (7/8) (Fig 2C'), there was one example of incomplete closure and this sample also exhibited extensive degradation of the P3 bone.

HBO treated digits also showed distinct changes within the marrow cavity prior to blastema formation. H&E analysis of HBO treated digits at 7 DPA showed more pitting on the endosteal side of the bone when compared to the periosteal side. Additionally, the marrow cavity itself displays other remarkable differences (Fig 2A and 2A'). One of the characteristics of the P3 marrow region in control digits was the presence of a single very large vessel filled with red blood cells that occupied much of the marrow space (Fig 2A, arrowhead). In HBO treated digits the marrow vasculature appears smaller and the marrow region itself is filled with loosely packed nucleated cells with spindle morphology (Fig 2A', arrows). These cells tested negative for CD45 suggesting that they are not involved in the inflammatory response. However, approximately half of the HBO treated digits analyzed at 7 and 10 DPA displayed an encapsulated CD45 positive cell mass that was not observed in control regenerates (S3 Fig). These data indicate that concomitant with degradation and immediately prior to blastema formation, the marrow of HBO treated animals undergoes a replacement of its predominantly hematopoietic cell content with a dramatic expansion of a non-hematopoietic lineage.

Close examination of the endosteal cell layer identified changes in response to daily HBO treatment relative to control endosteum. Inactive endosteal cells assume a flattened morphology but take on an activated cuboidal morphology during bone remodeling [1, 19, 20]. In digit regeneration both control digits and HBO treated digits demonstrated activated osteoblasts in the endosteal cell layer at 7 DPA (Fig 2A and 2A', insets). By 10 DPA all of the 8 control digit samples returned to a flattened inactive morphology (Fig 2B inset) in the marrow cavity and maintained this morphology throughout the remainder of the regeneration timeline (Fig 2C inset). Distal to the marrow cavity, at the edge of the amputation stump, normally squamous osteoblasts assumed a more activated cuboidal morphology. This indicates that during normal regenerative events endosteal osteoblast activation is transiently induced in the stump prior to blastema formation. In contrast, HBO treated digits displayed activated osteoblasts at both 10 (Fig 2B' inset) and 14 DPA (Fig 2C' inset), indicating that increased oxygen prolonged this transient period of endosteal activation. It is noteworthy that the extension of osteoblast activation correlated with the extended period of osteoclast activity associated with HBO treatment. This suggests that the well-studied interaction between osteoclasts and osteoblasts in the control of bone homeostasis is active during blastema formation and the epimorphic regenerative response.

**Fig 3:**
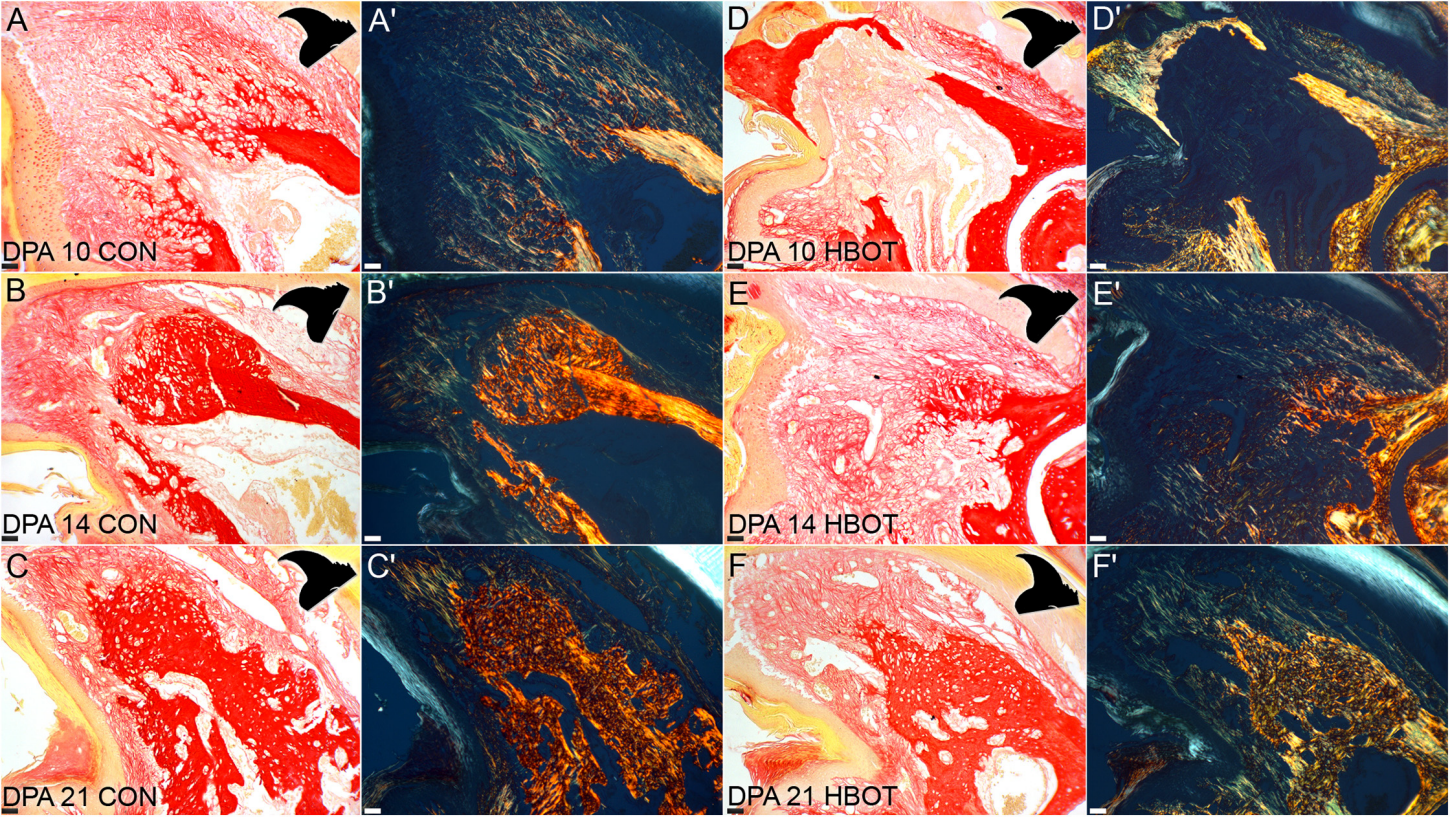
HBO treatment alters the morphology of regenerated bone. Polarized light micrographs of control and HBO treated digits at 7, 10, 14, and 21 DPA show differences in collagen fiber composition at distinct time points during regeneration. Collagen fiber alignment and thickness is readily identifiable under polarized light. Thinner fibers (a general indication of either thin collagen I fibrils, <50 nm in width, or collagen III fibers) appear green. Thick, more mature and aligned collagen I fibers appear red or yellow [22]. Photomicrographs are presented in transmitted light (A—F) and polarized light (A'-F'). Digit orientation is portrayed by black cartoon on the upper right corner of each image. Scale bars = 100 μm. N = 3 with representative sample shown.

Following the enhanced histolytic phase, a delayed blastema phase is evident as part of regeneration in HBO treated digits. Blastema formation in the control digit typically occurs at 10 DPA, is largely avascular and is located just distal to the marrow cavity (Fig 2B) [5, 16, 18]. Extracellular matrix analysis of the control blastema at 10 DPA using Picro-Sirius red staining [21] with (Fig 3A) and without (Fig 3A') polarized light showed low levels of mature collagen I (50 nm thick fibers appear yellow/red in Picro-Sirius red staining [22]), and abundant levels of thinner collagen fibrils at the interface between the digit stump and the proximal blastema. This is consistent with previous studies investigating the collagen composition of the digit blastema [16]. At 10 DPA HBO-treated digits had not completed the histolytic phase or wound closure, and the marrow space presented with small vasculature and high cell density (Fig 2B'). Interestingly, there was some evidence of new collagen deposition in the marrow space directly proximal to the dorsal/ventral gaps of degraded bone (Fig 3D and 3D'). These fibers are not observed at later time points when the marrow space is regenerated. At 14 DPA after complete wound closure, blastema formation was evident in HBO-treated digits. It is noteworthy that blastema formation was not only delayed but also extends to a more proximal site compared to controls. In HBO treated digits, the blastema formed where the marrow cavity once existed (Fig 2C') and there was no evidence of the large vessel seen in control digits at the same time point (Fig 2C). Instead collagen deposition was seen in the highly cellular region that occupied the former marrow space. In the blastemas of HBO treated digits as in control digits, thin collagen fibrils were more abundant than thick collagen fibrils (Fig 3D and 3D'). Thus, as with controls, wound closure and blastema formation were concurrent in HBO-treated digits, and the ECM composition of the blastema after HBO treatment is similar to that of controls.

### HBO treatment extends the level of regenerative competency and alters the morphology of the regenerated bone

**Fig 4:**
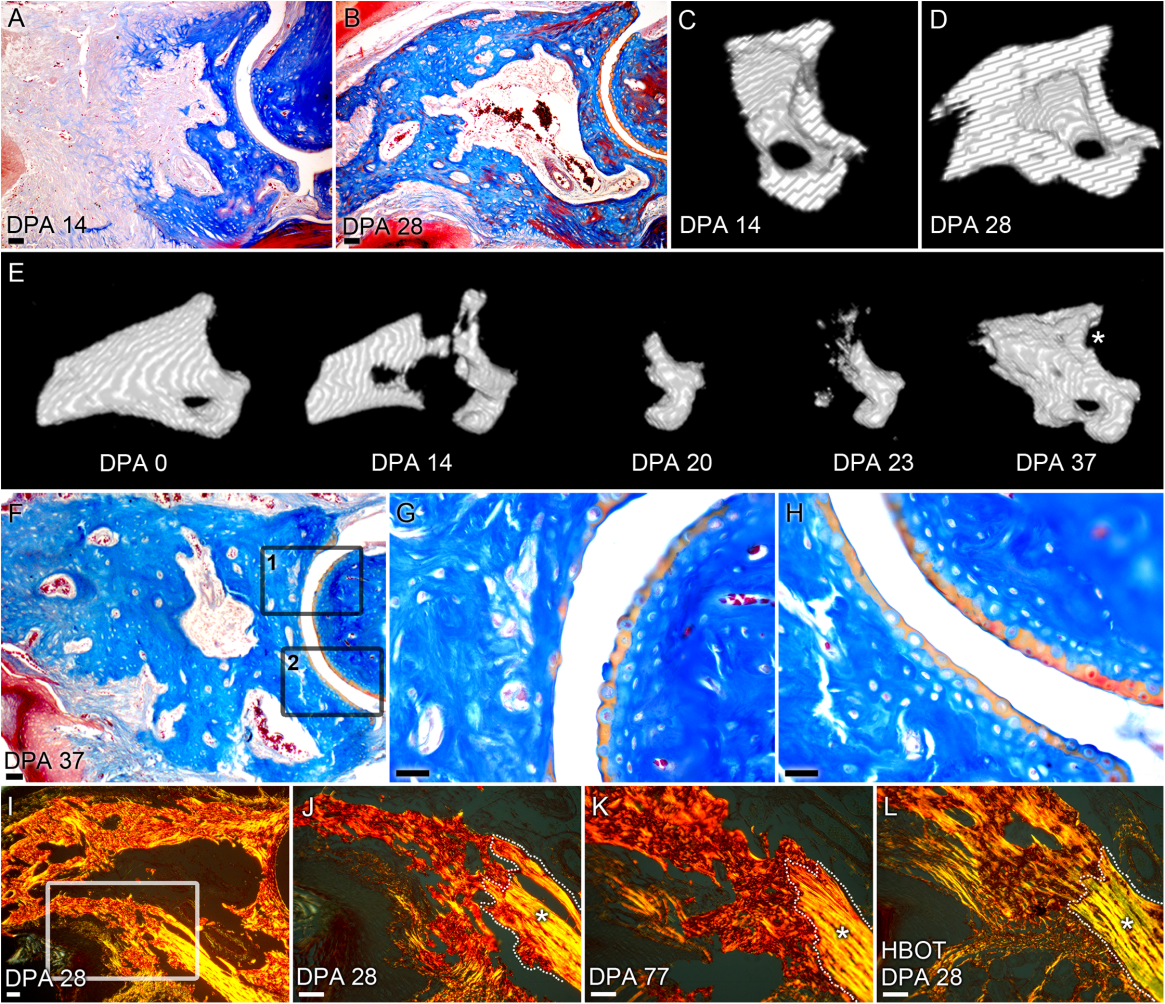
Hyperbaric oxygen extends the level of regenerative competency. A) Mallory trichrome staining at 14 and (B) 28 DPA of a HBO treated digit show proximal degradation of P3 bone and subsequent patterned bone and regeneration of the marrow cavity. Scale bar = 100 μm. Cross-sections of μCT-generated 3D renderings of the sample shown in 4B at 14 DPA (C) and 28 DPA (D) show regeneration of the marrow cavity and patterned distal outgrowth of bone. (E) μCT-generated 3D renderings show exacerbated bone degradation through the P3 joint area that regenerates along with the distal bone. The regenerated P3 shows a defect in the joint at DPA 37 (asterisk), which coincides with an imperfect joint lining in this area (G). (F) Mallory trichrome staining of the regenerated digit at 36 DPA shows imperfect joint patterning in the dorsal region of the joint (G, box 1 in F) when compared to a control P2/P3 joint (S4 Fig). Scale bar = 100 μm. Higher magnification images show regenerated, but discontinuous staining of the cartilage joint region when compared to the non-degraded ventral region (H, box 2 in F). Scale bar = 25 μm. (I and J) Picro-Sirius red staining of control digits at 28 DPA shows regenerated woven bone, that (K) do not resolve trabecular woven bone to lamellar bone even after 77 days. Scale bar = 50 μm. (L) In contrast, 28 DPA HBO treated digits show parallel collagen I fibers that are more akin to the lamellar bone seen in the cortical bone stump. Scale bar = 50 μm. Original lamellar bone stump is outlined and indicated by an asterisk, highlighting the boundary with the newly regenerated bone. (N = 3, representative figure shown).

As noted earlier, digits lose a larger bone mass with HBO treatment and blastema formation is delayed. The ability of digits to regenerate following treatment was analyzed. We found that regrowth of P3 pattern was maintained in all digits despite the oxygen enhanced level of degradation, and noted that in 9 of 16 digits a successful regenerative response occurred from proximal-distal levels that are typically non-regenerative [11]. Correlating μCT imaging and histological analyses (Fig 4B, 4C and 4D, same sample; Fig 4A, representative sample) provide evidence that the marrow cavity can degrade and reform in HBO treated regenerates. In one extreme example, μCT analyses documented an oxygen-induced degradation response that eroded approximately 90% of the original amputated P3 bone volume, yet successfully transitioned into a regenerative response. This sample displayed degradation into the dorsal region of the P2/P3 joint, and completely eroded the ventrally located os-hole. Subsequent μCT imaging of this sample showed the regeneration of the digit tip including the P3 bone of the P2/P3 joint and the os-hole (Fig 4E). μCT imaging of the digit shows the complete degradation of the dorsal P2/P3 joint area (Fig 4E, DPA 20). μCT scans from 37 DPA show a "C"-shaped concavity in the regenerated bone (Fig 4E, DPA 37, asterisk). Histological analysis of the regenerated digit confirms that the Mallory trichrome staining of the joint cartilage is discontinuous and less organized along this dorsal section of the joint (Fig 4G) when compared to the uncompromised ventral section of the same joint (Fig H). These data are suggestive that the degradation response compromised the bone as well as the cartilage of the P3 joint area yet the joint subsequently regenerated, albeit imperfectly. This example provides evidence that even from a very proximal level of P3, the digit is able to mount a regenerative response that includes appropriately patterned bone as well as a new marrow cavity.

Picro-Sirius red staining and polarized light microscopy revealed differences in the morphology of the regenerated bone. Control digits began to regenerate bone at 14 DPA and showed the characteristic woven trabecular bone (Fig 3B and 3B'). While HBO treated digits showed blastema formation at the same time point (Fig 3E and 3E'), bone formation at 21 DPA revealed a marrow cavity devoid of collagen fibers (Fig 3F and 3F'). Evidence of a collagen-free marrow cavity showed that the presence of collagen fibers seen in the marrow cavity at 14 DPA is a transient event. Towards the endpoint of regeneration, the bone of regenerated HBO treated digits showed more organized patterning of collagen fibers. High magnification polarized light analysis of a regenerated control digit at 28 DPA highlighted the difference between the parallel collagen I fibers consistent with lamellar bone in the original bone stump and the newly regenerated bone characterized by a woven pattern of collagen more akin to trabecular bone formation (Fig 4I and 4J). In contrast to the woven bone seen in control digits (Fig 3C and 3C') and also the woven bone seen in HBO treated digits at 21 DPA (Fig 3F and 3F'), HBO treated samples at 28 DPA showed remodeled bone that is more akin to lamellar bone where collagen fibers are parallel and assembled in a pattern comparable to that of the original bone stump (Fig 4L). Interestingly, control digits do not remodel into more lamellar-like bone and show woven bone patterning even at 77 days post-amputation (Fig 4K). Taken together these data show that HBO treated digits are able to form a proximal blastema that maintains a blueprint of the P3 skeletal structure, successfully regenerate from a very proximal level, and ultimately remodel the regenerated bone into cortical bone.

## Materials and Methods

All experiments were performed in accordance with the approved standard operating procedures by the Institutional Animal Care and Use Committee of Tulane University Health Sciences Center. All procedures were approved by the Tulane University IACUC Office. Anesthetics: Isoflourane gas—Admnistered with an isoflourane precision vaporizer (VetEquip). 2–5% isoflourane (in a chamber) is used to anesthetize the mouse, and 2% isoflourane (nose cone) is used to maintain anesthesia during the surgery. Ketamine/Xylazine (working solution, 90.9 mg/ml ket, Xyl 9.1 mg/ml; 0.9 ul/g body weight; IP) is used to anesthetize adult mice (approved). No post-surgical analgesia was required. Euthanasia overdose with Ketamine/Xylazine (7.0ul/g body weight) followed by cervical dislocation (approved). Protocol #0311R-UT

### Amputations and animal handling

Adult 8-week old [4] female CD1 mice were obtained from Charles River Laboratories (Wilmington, MA). Mice were anesthetized with 1–5% isoflurane gas with continuous inhalation. The second and fourth digits of both hind limbs were amputated at the P3 distal level as described previously [1, 5] and regenerating digits were collected at designated times for slice culture. The third digit was used as an unamputated control. All experiments were performed in accordance with the standard operating procedures approved by the Institutional Animal Care and Use Committee of Tulane University Health Sciences Center.

### Tissue collection and histology

Digits were fixed overnight in zinc-buffered formalin (Z-fix, Anatech, Battle Creek, MI). Bone was decalcified for 8 hours in a formic-acid based decalcifier (Decal I, Surgipath, Richmond, IL). Once decalcified, all samples were processed for paraffin embedding using a Leica TP 1020 Processor (Leica, Buffalo Grove, IL) and sectioned at 4 microns onto glass slides. Sections were stained with either Mayer’s Hematoxylin and Eosin Y, Mallory trichrome, or Picro-Sirius red stain (Sigma-Aldrich, St. Louis, MO) and mounted using permanent mounting medium (Fisher Scientific, Waltham, MA). Brightfield micrographs were captured using an Olympus DP72 camera mounted on an Olympus BX60 microscope with rotating stage (Olympus America Inc, Center Valley, PA). Polarized light microscopy was carried out with filters to provide circularly polarized illumination according to published methods [21]. Final figures were generated using Adobe Photoshop CS4.

### Fluorescent immunohistochemistry

Immunofluorescent staining was performed on deparaffinized and rehydrated sections with specific primary antibodies: CD45 (Abcam, Cambridge, MA) (ab10558; 1 μg/mL). Primary signal was amplified with Alexa Fluor secondary antibodies (Invitrogen, Carlsbad, CA) and analyzed for bone formation using an epifluorescence deconvolution microscope (Olympus America Inc, Center Valley, PA).

### Radiography and micro-computed tomography (μCT)

Digit X-rays were taken with a Kodak In-Vivo Imaging System FX (Carestream, Rochester, NY) using the standard settings (35 kV for 60 s). Micro-CT images were acquired using a VivaCT 40 (Scanco Medical AG, Brüttisellen, Switzerland) at 1000 projections per 180 degrees with a voxel resolution of 10 μm^3^, and energy and intensity settings of 55 kV and 145 μA. Integration time for capturing the projections was set to 380 ms using continuous rotation. Images were segmented using the BoneJ [23] (Version 1.3.11) Optimize Threshold Plugin for ImageJ (Version 1.48s). Changes in bone volume and trabecular thickness were quantified using the BoneJ Volume Fraction Plugin for ImageJ and bone volume was normalized to total bone volume at DPA 0. Changes in bone thickness were mapped using the Trabecular Thickness Plugin for ImageJ and 3D renderings of the μCT scans and the bone thickness maps were created using the 3D viewer plugin for ImageJ.

### Hyperbaric Oxygen Treatment (HBOT)

The control group did not receive HBO treatment. Mice were placed in a hyperbaric chamber (Baromedical Research Institute—Van Meter and Assoc., Harvey, LA) and received 90 min of 100% oxygen at 2.4 atmospheres absolute (ATA). Application was three times daily for the first two days and twice daily for each day after for a total of 28 days of treatment. Compression and decompression of the chamber were executed at 2 psi/minute.

### Statistical analysis

Quantitative group differences were determined using a two-tailed Fisher’s exact test for different groups at the same time point. A value of p<0.05 was deemed statistically significant. In all cases, data are represented as mean ± standard error of mean (SEM). Statistically significant differences for NOc/BPm were determined using unpaired t-tests with two-tailed distributions using KaleidaGraph (Version 4.1.1, Synergy Software, Reading, PA). A value of p<0.05 was deemed statistically significant. In all cases, data are represented as mean ± standard error of mean (SEM).

### Smoothing Spline ANOVA

Volume measurements for each digit were analyzed for the 28-day time period following amputation, with measurements expressed in terms of the volume percentage relative to the post-amputation baseline. Mixed effect Smoothing Spline Analysis of Variance (SS ANOVA) models [24, 25] were fit to the collected data to account for the nonlinearity of the growth trajectories and correlation in the repeated measurements for each animal and amputated digit. The R package ASSIST [26](Version 3.1.2), was used to estimate curves and approximate Bayesian 95% confidence intervals for the average growth levels in each treatment group.

## Discussion

The regenerating mouse digit has become a premiere model for epimorphic regeneration in mammals. Using this model we have demonstrated that the regenerative process itself constitutes a dynamic series of oxygen events, and have shown that targeted modification of oxygen events significantly alters phase transitions critical for the regenerative response [1, 16]. Here we expand upon these findings by applying a daily application of HBO treatment and show that continuous oxygen exacerbates bone degradation shifting the level of regeneration toward the P3 joint. Whereas, endogenous regenerative responses do not normally occur from such proximal amputation levels [27–29], regeneration readily occurs in these HBO treated digits. Remarkably, we document complete regeneration of the P3 bone marrow, patterned regeneration of a distinct skeletal structure (os-hole), and evidence for the endogenous regeneration of joint tissue. Additionally, regenerated bone displays composition changes that suggest a role for oxygen treatment in the deposition of collagen fibers that are more highly organized and compact. These data provide further support for the general conclusion that the dynamics of oxygen is a key player in the regulation of an epimorphic regenerative response in mammals.

The daily application of hyperbaric oxygen dramatically extends the duration and degree of the bone degradation phase of digit regeneration. Osteoclast activity is closely linked to bone degradation during regeneration [5], and exacerbated bone degradation by HBO treatment is associated with a prolonged period of osteoclast activity. The osteoclast is a highly specialized multinucleated cell type derived from monocytes of the hematopoietic system that functions in the local erosion of bone tissue. In bone homeostasis, it is well known that osteoclast activity is intricately linked to osteoblast activation [30], and we observe a similar enhancement of osteoblast activation during digit regeneration. Since osteoprogenitor cells are known to contribute to the blastema and to regenerated bone during digit regeneration [31, 32], this evidence suggests that osteoclast activity not only is key in histolysis of stump bone tissue but also activates osteoblasts associated with the bone stump that can participate in blastema formation. This conclusion is consistent with the demonstration in other injury models that osteoclastogenesis and osteoclast activation triggers proliferation and mobilization of stem cells associated with the endosteal niche of the bone marrow [33, 34]. The role of osteoclast activity in activating and releasing resident blastema progenitor cells also provides an explanation for how regenerative ability can be maintained following degradation of the stump proximally into regions of the digit that are normally regeneration-incompetent.

While osteoclast activity and the degradation of bone tissue in the stump is a very visible and quantifiable structural outcome, soft tissues of the stump are likely to undergo histolysis in parallel. Numerous studies have identified the up-regulation of enzymes known to degrade extracellular matrix components during regeneration of the amphibian limb as well as the mouse digit [35–37]. In addition, modulation of matrix degrading enzymes can influence wound closure and regenerative outcome in amphibians [38, 39] and, more recently, stimulate soft tissue regenerative responses in mice [40]. Bioactive cryptic peptides derived from a degraded ECM scaffold preparation has been shown to enhance stem cell recruitment and osteogenesis after digit amputation [41, 42], and enhanced cell recruitment plays a key role in endogenous and BMP2 stimulated regenerative responses [43]. Collectively, these studies are consistent with the idea that histolysis of stump tissues is a crucial early step in creating a blastema-permissive wound site, and mobilizing resident blastema progenitor cells that can participate in blastema formation. This conclusion is further supported by experimental studies showing that a reduced osteoclast presence leads to a decrease in bone degradation, and a visibly smaller blastema [16], whereas, our current finding shows that HBO enhanced osteoclast activity results in the formation of enlarged blastemas. Thus, blastema size, and presumably progenitor cell availability, appears to be intricately linked to the level of histolytic activity in the stump.

The regenerating mouse digit is characterized by a relatively slow and tortuous wound closure response. After amputation, the wound epidermis does not close over the stump bone but instead epidermal closure is delayed while the stump bone undergoes an osteoclast driven re-amputation of the stump [5]. Wound closure occurs by the migration of the wound epidermis through the re-amputation, the distal bone fragment is cast off, a blastema forms between the wound epidermis and osteoclast activity is no longer evident in the amputation wound. This type of coordination between osteoclast activity and epidermal closure is not unique to the regenerating mouse digit but is also observed in antler casting prior to antler bud formation during antler regeneration [44]. In a recent digit regeneration study that used a cyanoacrylic wound dressing (Dermabond) known to increase the rate of epidermal closure, we showed that rapid epidermal closure was associated with hypoxic epidermal cells and a decrease in post-amputation bone loss, and that HBO treatment reverses this effect [16]. Here we show that continuous HBO treatment alone causes a further delay in wound closure that correlates with the extended period of osteoclast activity, thus the data suggest a link between epidermal closure and the termination of histolysis in digit regeneration. One possibility is that the oxygen level coordinates the activities of multiple cell types and in different tissues of the stump to successfully organize a complex regenerative response. An alternative, but not mutually exclusive, idea is that feedback interactions between different cell types that are responsive to oxygen help to facilitate phase transitions, such as the switch from histolysis to blastema formation, important for the regenerative response. The existence of such feedback interactions help to reconcile differences in the effect of HBO treatment on epithelialization rates in other wound healing models [45, 46].

HBO is currently established for many clinical applications and has potential for use in regenerative therapy, yet its mechanism of action in most applications remains unknown. The application of HBO treatment described here is similar to the daily application that is the standard of clinical care recommended by the UHMS and offers the first comprehensive investigation of the effects of HBO on epimorphic regeneration. One long-term goal of this effort is to optimize the timing and dosage of HBO in order to exploit oxygen as a potential proactive force in human regeneration. In combination with previous studies on digit regeneration [1, 16], the evidence supports the idea that after identifying a target effect within regeneration, it is feasible to adjust the timed application of HBO to effect a desired outcome. Khanna et al demonstrated *in vitro* that it is possible to reset the normoxic set point of the oxygen-sensing hypoxia-inducible factor [47], thus daily application of HBO is likely to have differential effects on different cell types and systems depending on their oxygen set-point. Along a similar line of reasoning, oscillating oxygen tensions by targeting specific time points during regeneration [1], is likely to effect target outcomes without resetting the normoxic set point. Future studies that further dissect the existing relationships between the heightened bone degradation, conferred proximal regenerative capability, and positive effects on bone morphology present a break-through opportunity to both understand and control the regenerative capacity. Taken together, these data provide strong evidence that applied oxygen treatment has the potential to significantly enhance regenerative capability in mammals.

## Supporting Information

S1 Fig Hyperbaric oxygen amplifies timing and volume variability between digits during regeneration. (A) Tracking the bone volume changes of individual digits during the regeneration process reveals increased variability between individual digits during regeneration. Control and HBO treated samples shown individually. Samples were analyzed for bone growth using μCT. Data are normalized to initial DPA 0 bone volume. (N = 4 mice, N = 16 digits). (B) Traditional grouping of digits by time point with use of error bars. T-tests between time points assumes independence at each time point as well as independence of digits within mice and inflates the probability of false-negative results. Control and HBO treated samples shown individually. Samples were analyzed for bone growth using μCT. Data are normalized to initial DPA 0 bone volume. (N = 4 mice, N = 16 digits). Results are expressed as mean ± SEM. ^#^ P<0.05, comparison of control to HBO, where time points are comparable.
(TIF)

S2 Fig Unamputated digits show no bone degradation or histological changes after HBO treatment. (A) HBO treated unamputated digits show no adverse effects or substantive remodeling after daily treatment with HBO. H&E staining shows no histological differences between (B) unamputated digits that are not treated with HBO and (C) unamputated digits that are treated with HBO at day 28. Samples were analyzed for bone growth using μCT. Data are normalized to initial unamputated bone volume. (N = 4 mice, N = 16 digits). Scale bar = 100 μm. Results are expressed as mean ± SEM.
(TIF)

S3 Fig Digits treated with hyperbaric oxygen show CD45-positive cell mass. HBO treated digits are positive for CD45 staining in the encapsulated cell mass, and minimal signal in surrounding areas. (A) H&E staining and (B) CD45 positive staining of a serial section of HBO treated digit at DPA 10 (shown in Fig 2). Red = CD45, Grey = Nuclei. Scale bar = 50 μm. N = 3 with representative sample shown.
(TIF)

S4 Fig Representative P2/P3 joint from a control digit. Untreated control digits stained by Mallory trichrome showed continuous joint cartilage (yellow) with organized chondrocyte zones. Scale bar = 25 μm.
(TIF)

S1 Movie Time lapse imaging of a representative digit (from A) showing the P3 regenerative response in a control digit. (AVI)

S2 Movie Time lapse imaging of a representative digit (from A) treated with HBO showing prolonged bone degradation and a slower rate of bone growth. (AVI)
