# Supplementary figures and images for: Hyperbaric Oxygen Promotes Proximal Bone Regeneration and Organized Collagen Composition during Digit Regeneration
Source: PLoS One. 2015 Oct 9;10(10):e0140156. doi: 10.1371/journal.pone.0140156 (PMC4599941; doi:10.1371/journal.pone.0140156)

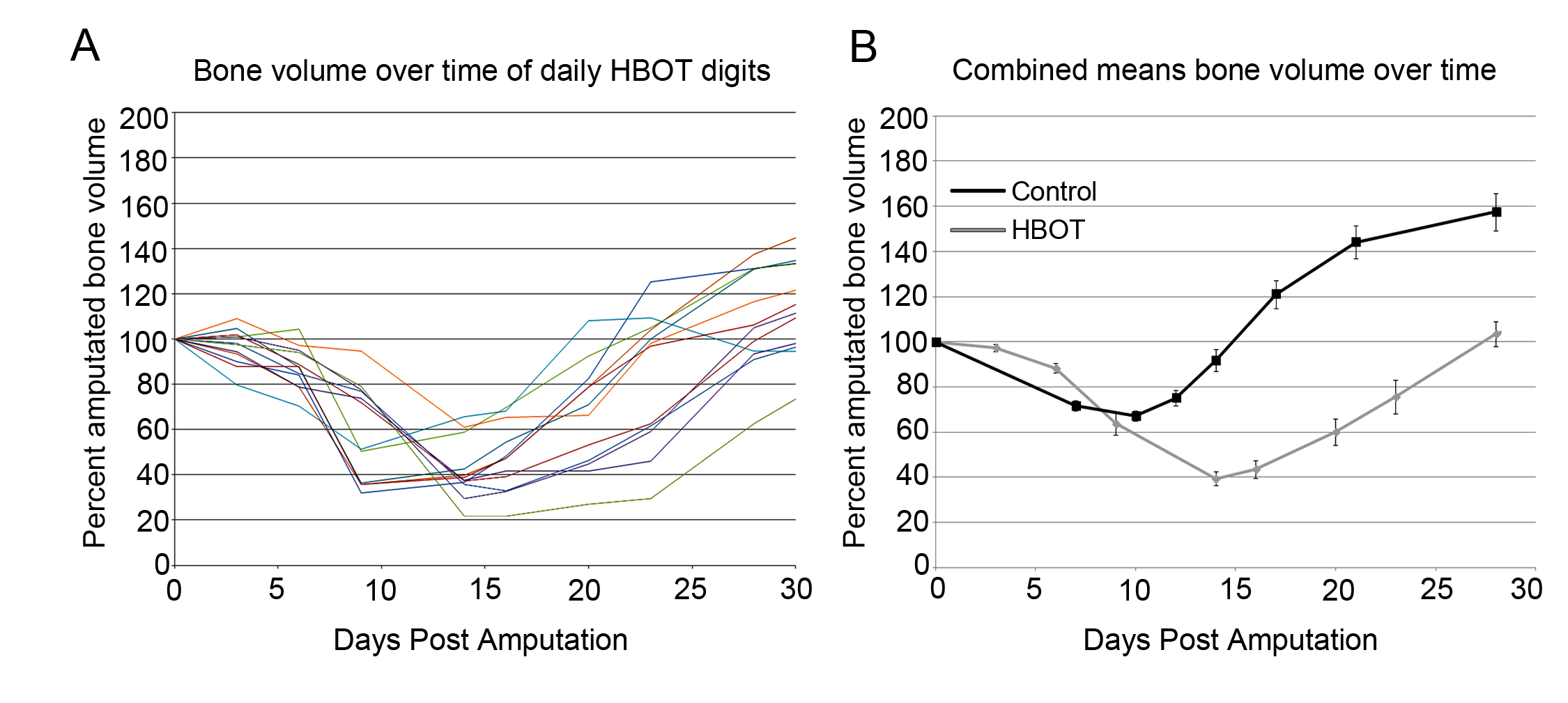

Supplement: S1 Fig — (A) Tracking the bone volume changes of individual digits during the regeneration process reveals increased variability between individual digits during regeneration. Control and HBO treated samples shown individually. Samples were analyzed for bone growth using μCT. Data are normalized to initial DPA 0 bone volume. (N = 4 mice, N = 16 digits). (B) Traditional grouping of digits by time point with use of error bars. T-tests between time points assumes independence at each time point as well as independence of digits within mice and inflates the probability of false-negative results. Control and HBO treated samples shown individually. Samples were analyzed for bone growth using μCT. Data are normalized to initial DPA 0 bone volume. (N = 4 mice, N = 16 digits). Results are expressed as mean ± SEM. # P<0.05, comparison of control to HBO, where time points are comparable. (TIF) [file pone.0140156.s001.tif]

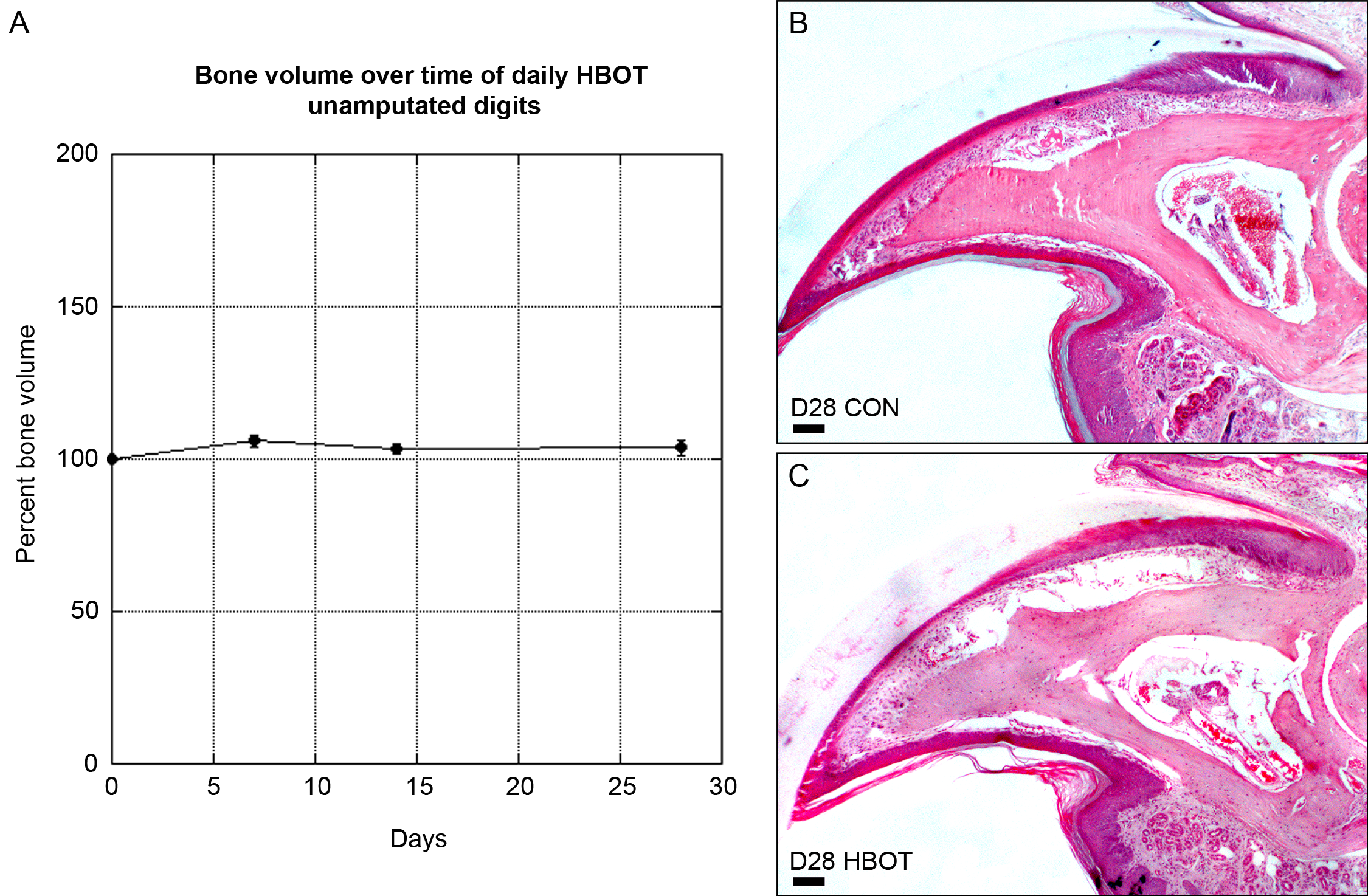

Supplement: S2 Fig — (A) HBO treated unamputated digits show no adverse effects or substantive remodeling after daily treatment with HBO. H&E staining shows no histological differences between (B) unamputated digits that are not treated with HBO and (C) unamputated digits that are treated with HBO at day 28. Samples were analyzed for bone growth using μCT. Data are normalized to initial unamputated bone volume. (N = 4 mice, N = 16 digits). Scale bar = 100 μm. Results are expressed as mean ± SEM. (TIF) [file pone.0140156.s002.tif]

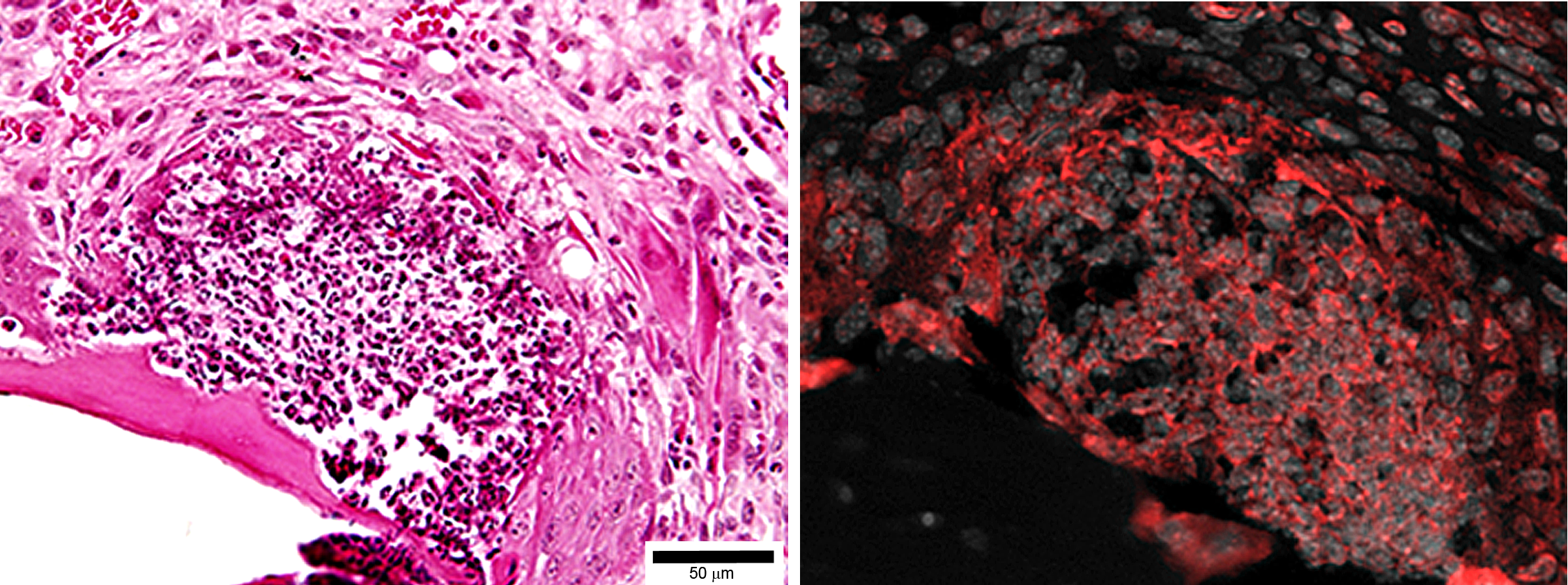

Supplement: S3 Fig — HBO treated digits are positive for CD45 staining in the encapsulated cell mass, and minimal signal in surrounding areas. (A) H&E staining and (B) CD45 positive staining of a serial section of HBO treated digit at DPA 10 (shown in Fig 2). Red = CD45, Grey = Nuclei. Scale bar = 50 μm. N = 3 with representative sample shown. (TIF) [file pone.0140156.s003.tif]

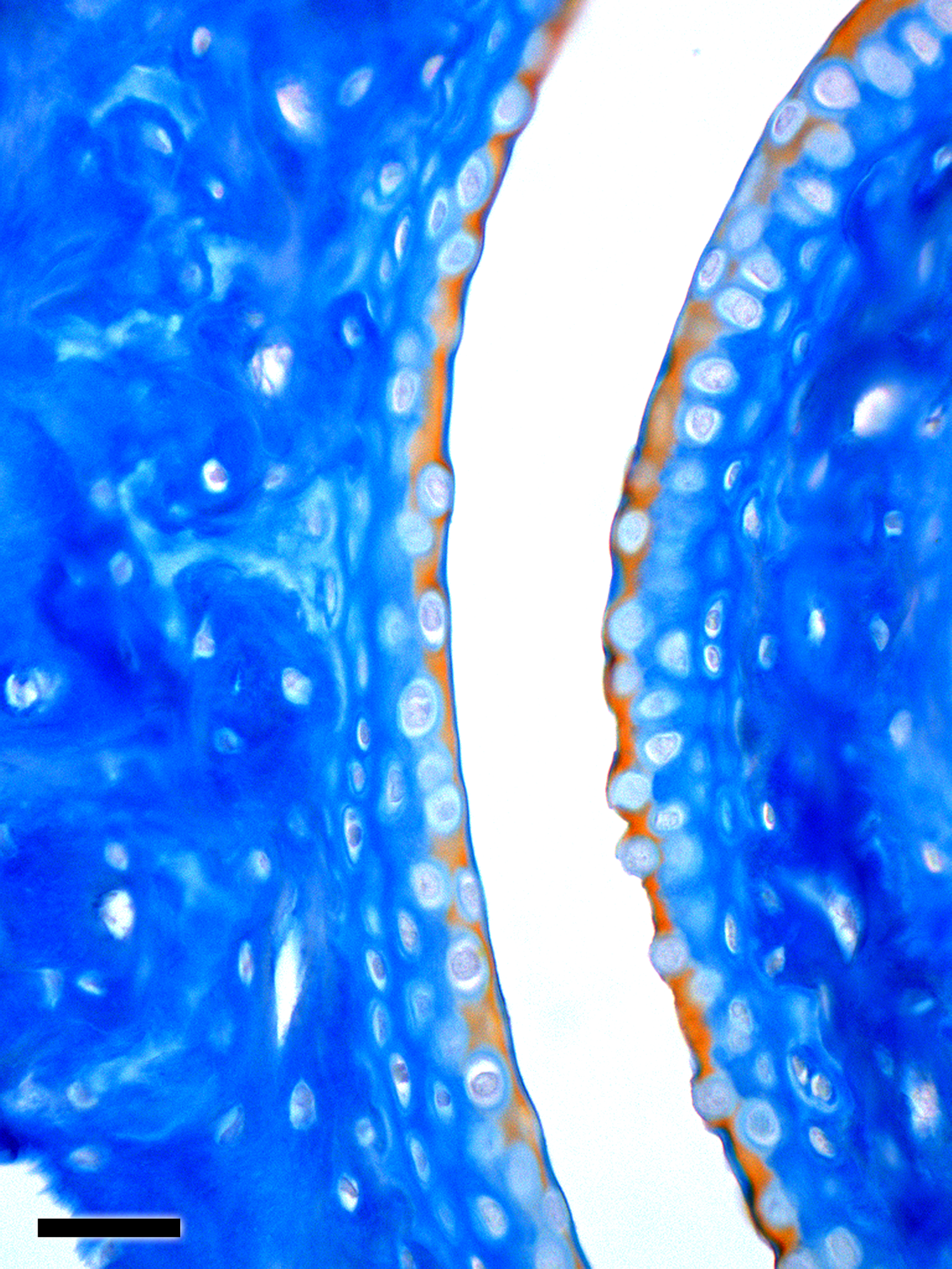

Supplement: S4 Fig — Untreated control digits stained by Mallory trichrome showed continuous joint cartilage (yellow) with organized chondrocyte zones. Scale bar = 25 μm. (TIF) [file pone.0140156.s004.tif]
